# A Bioengineered Nisin Derivative, M21A, in Combination with Food Grade Additives Eradicates Biofilms of *Listeria monocytogenes*

**DOI:** 10.3389/fmicb.2016.01939

**Published:** 2016-11-30

**Authors:** Muireann K. Smith, Lorraine A. Draper, Pieter-Jan Hazelhoff, Paul D. Cotter, R. P. Ross, Colin Hill

**Affiliations:** ^1^APC Microbiome Institute, School of Microbiology, University College CorkCork, Ireland; ^2^Wageningen University & ResearchWageningen, Netherlands; ^3^Teagasc Food Research CentreCork, Ireland

**Keywords:** *Listeria monocytogenes*, biofilm, nisin, bioengineered, citric acid, cinnamaldehyde, combinations

## Abstract

The burden of foodborne disease has large economic and social consequences worldwide. Despite strict regulations, a number of pathogens persist within the food environment, which is greatly contributed to by a build-up of resistance mechanisms and also through the formation of biofilms. Biofilms have been shown to be highly resistant to a number of antimicrobials and can be extremely difficult to remove once they are established. In parallel, the growing concern of consumers regarding the use of chemically derived antimicrobials within food has led to a drive toward more natural products. As a consequence, the use of naturally derived antimicrobials has become of particular interest. In this study we investigated the efficacy of nisin A and its bioengineered derivative M21A in combination with food grade additives to treat biofilms of a representative foodborne disease isolate of *Listeria monocytogenes*. Investigations revealed the enhanced antimicrobial effects, in liquid culture, of M21A in combination with citric acid or cinnamaldehyde over its wild type nisin A counterpart. Subsequently, an investigation was conducted into the effects of these combinations on an established biofilm of the same strain. Nisin M21A (0.1 μg/ml) alone or in combination with cinnamaldehyde (35 μg/ml) or citric acid (175 μg/ml) performed significantly better than combinations involving nisin A. All combinations of M21A with either citric acid or cinnamaldehyde eradicated the *L. monocytogenes* biofilm (in relation to a non-biofilm control). We conclude that M21A in combination with available food additives could further enhance the antimicrobial treatment of biofilms within the food industry, simply by substituting nisin A with M21A in current commercial products such as Nisaplin^®^ (Danisco, DuPont).

## Introduction

The globalization of food distribution and human travel (Tauxe, 2002; Hussain and Dawson, 2013) has meant that foodborne disease can spread further and more rapidly than ever before, bypassing conventional control measures (Koopmans et al., 2003; World Health Organization and Foodborne Disease Burden Epidemiology Reference Group, 2007). The ability of bacterial pathogens to remain viable in food production environments, even with the enforcement of stringent hygiene practices, can be in part due to the accumulation of resistance mechanisms (Holah et al., 2002; Tauxe, 2002) and through the protection provided via the formation of biofilms (Borucki et al., 2003; Ryu and Beuchat, 2005; Houdt and Michiels, 2010). Surface-attached biofilms are sessile microbial communities that can be irreversibly attached to a substratum and are enclosed in an extracellular polymeric matrix (Donlan, 2002; Olszewska, 2013; Colagiorgi et al., 2016). Once formed, biofilms can be extremely difficult to remove, leading to contamination of processing equipment (Kumar and Anand, 1998; Tan et al., 2014), food spoilage and consequent economic losses (Brooks and Flint, 2008; Houdt and Michiels, 2010). Numerous studies have described the use of novel approaches to control biofilms; these have included the use of biocides (Wood et al., 1996), application of electrical currents (Poortinga et al., 2001), temperature step changes during growth regions (Knight et al., 2004), enzymes (Liu et al., 2014) and for biofilms on or within a food matrix, the use of novel food additives (Furukawa et al., 2010).

The use of bio-preservatives, as an alternative to chemical food preservatives, has become more popular due to customer demand for more natural and minimally processed foods (Lucera et al., 2012). One such group of anti-bacterial preservatives are bacteriocins. Bacteriocins are small, ribosomally synthesized antimicrobials (Cleveland et al., 2001; Cotter et al., 2005; Gálvez et al., 2007; Yang et al., 2014). Those derived from lactic acid bacteria (LAB) have gained particular attention with respect to their use in the food industry. One of the most extensively studied bacteriocins is nisin A, a bactericidal lantibiotic produced by some strains of *Lactococcus lactis* (Rouse et al., 2012; Campion et al., 2013; Healy et al., 2013). It is a 3.5 kDa peptide consisting of 34 amino acids and is a flexible and elongated amphipathic peptide that has the ability to form pores as well as inhibit cell wall biosynthesis (Breukink et al., 1999; Wiedemann et al., 2001; Prudêncio et al., 2015). This dual action of nisin has meant that despite widespread use in the food industry, there have been few reports of resistance development outside of the laboratory (Field et al., 2010b; Molloy et al., 2012). Due to its proven range of antimicrobial effects, nisin has been used as a food preservative for many years (Breukink and de Kruijff, 1999; Elliason and Tatini, 1999; Gálvez et al., 2007). Nisaplin^®^ is an example of a commercially available food additive, containing 2.5% nisin. It is Generally Regarded As Safe (GRAS) for several applications and has been approved for use in the EU as preservative E234 by Directive 95/2/EC (European Food Safety Authority, 2006) by the U.S. Food and Drug Administration (FDA) (Prudêncio et al., 2015) and by the WHO (Sobrino-López and Martín-Belloso, 2008).

Other examples of food additives are organic acids such as lactic acid and citric acid (Young and Foegeding, 1993; Helander and Mattila-Sandholm, 2000; Norhana et al., 2012), salts of organic acids such as trisodium phosphate, potassium sorbate, sodium benzoate and sodium diacetate (Phillips and Duggan, 2001; Norhana et al., 2012) and proteins such as lactoferrin (Branen and Davidson, 2004; Gálvez et al., 2007) and nisin. Enhanced antimicrobial activity has been successfully demonstrated against Gram-positive pathogens when several of these antimicrobials were used in combination with nisin (Phillips, 1999; McEntire et al., 2003; Branen and Davidson, 2004; Bari et al., 2005; Murdock et al., 2007; Norhana et al., 2012). Plant essential oils are another group of additives that have gained increased attention due to a long history of use for their antimicrobial effects and the increased demand for more “natural” food products (Helander et al., 1998; Burt, 2004; Bakkali et al., 2007; Prudêncio et al., 2015). Essential oils are natural, volatile liquids obtained from plant material (Gálvez et al., 2007) that have aromatic qualities (Burt, 2004). A number of studies have also been carried out that demonstrate the enhanced antimicrobial activity of nisin in combination with essential oils such as cinnamaldehyde (Field et al., 2015a) and carvacrol (Pol and Smid, 1999) against a broad range of pathogens including Gram-positive organisms, such as *Listeria monocytogenes* and *Bacillus cereus* (Pol and Smid, 1999; Field et al., 2015a), and Gram-negative bacteria, including *Salmonella enterica* and *Escherichia coli* (Phillips and Duggan, 2001; Govaris et al., 2010; Norhana et al., 2012; Prudêncio et al., 2015).

The use of nisin in combination with other food preservatives provides a hurdle approach to controlling pathogens while reducing/removing the need for chemically synthesized preservatives (Chalón et al., 2012). These benefits can be further augmented through the use of bioengineered forms of nisin. The gene encoded and ribosomally synthesized nature of nisin has meant that nisin is a perfect candidate for bioengineering, in order to enhance the functionality of the peptide (Field et al., 2008; Molloy et al., 2012, 2013). Indeed, although there are numerous studies that demonstrate the enhanced inhibitory activity of nisin when used in combination with antimicrobial food additives, there is little information pertaining to the use of bioengineered nisin in combination with food additives to inhibit pathogens within the food industry. Here, we demonstrate that the use of a bioengineered derivative of nisin in combination with a series of natural food additives results in enhanced antimicrobial effects against *L. monocytogenes*, including relatively antimicrobial resistant biofilms of this problematic food borne pathogen.

## Materials and Methods

### Growth of Bacterial Strains

*Listeria monocytogenes* F6854 strain, linked to a single case of human illness traced to contaminated turkey frankfurters in 1988 (Nelson et al., 2004), was grown in Tryptic Soy Broth (TSB) (Merck, Germany) or Tryptic Soy Agar (TSB substituted with 1.5% w/v agar) at 37°C. *L. lactis* strains, *L. lactis* NZ9700, *L. lactis* NZ9800-pCI372 Nisin A, *L. lactis* NZ9800-pCI372 M21A (Field et al., 2008), *L. lactis* NZ9800–pCI372 AAA (Healy et al., 2013) and *L. lactis* NZ9800-pCI372 Nisin V (Rouse et al., 2012), were grown on M17 supplemented with 5% glucose (GM17) broth (Oxoid, England) or GM17 agar (GM17 broth substituted with 1.5% w/v agar) at 30°C. Strains were stocked in 40% glycerol and stored at -20°C.

### Deferred Antagonism Agar Diffusion Assay

Agar diffusion assays was performed in triplicate as previously described by Healy et al. (2013). Briefly, *L. lactis* strains producing nisin A, M21A, AAA and Nisin V were spotted onto a GM17 agar plate and allowed to grow overnight. The plate was then subjected to UV radiation for 30 min and then overlaid with GM17 soft agar (0.75% w/v agar) seeded with 2% overnight of *L. monocytogenes* F6854 and allowed to grow overnight at 37°C. Antimicrobial efficiency was determined through measurement of the zone of inhibition using digital calipers.

### Nisin Peptide Purification

Purification of nisin peptide was performed as described previously by Campion et al. (2013). Briefly, TY broth (tryptone, yeast extract, MnSO_4_H_2_0, MgSO_4_) was passed through a column (Length 70 cm, diameter 5 cm) one third packed with Amberlite XAD-16 beads (Sigma). Resulting broth was dispensed in 900 ml volumes and autoclaved. Overnight cultures of nisin producing strains *L. lactis* NZ9800 – pCI372 M21A (for M21A production) or *L. lactis* NZ9700 (for nisin A) were used to inoculate previously filtered TY broth (2 × 900 ml) supplemented with 20% glucose and 20% β-glycerophosphate. After overnight incubation at 30°C, the culture was centrifuged at 8500 *g* at 4°C for 20 min (Thermo Scientific, Sorvall^TM^ RC 6+ Centrifuge, USA). Culture supernatant was passed dropwise through a column containing 60 g Amberlite XAD-16 beads and discarded. Following a 500 ml wash of 30% ethanol the nisin was eluted from the column using 500 ml of 70% isopropanol (Fisher Scientific, UK) supplemented with 0.1% trifluoroacetic acid (TFA). Concurrently bacterial cell pellets were resuspended in 300 ml 70% isopropanol – 0.1% TFA. Following stirring at room temperature for 3 h the suspension was centrifuged at 8500 *g* at 4°C for 15 min and supernatant was retained. The column eluant was pooled with the post-centrifugation supernatant and concentrated to a volume of 300 ml using rotary evaporation (BÜCHI Rotavapor R-205, Switzerland). The pH of the sample was adjusted to pH 4.0 and it was subsequently passed through a 60 ml Strata C-18 E column (Phenomenex) that was previously washed with 60 ml methanol (Fisher Scientific, UK) and 60 ml H_2_O. After applying 120 ml 30% ethanol, nisin was eluted from the column using 60 ml 70% isopropanol – 0.1% TFA and stored at 4°C. For HPLC purification 10 ml volumes were concentrated to a volume of ∼2 ml by rotary evaporation and injected into a HPLC system (Shimadzu) equipped with a Phenomenex C12 Reverse-Phase (RP) column (Jupiter 4 μ proteo 90 Å, 250 mm × 10.0 mm, 4 μm). Nisin was eluted via a gradient of 30–50% acetonitrile (Fisher) containing 0.1% TFA that was developed over 5–40 min. Nisin containing fractions were pooled, acetonitrile was removed by rotary evaporation and the peptide was lyophilised and stored at -20°C.

### Minimum Inhibitory Concentration Determination

Minimum inhibition concentrations (MIC) of peptides and antimicrobials were determined using 96 well microtitre plates following CLSI (Clinical and Laboratory Standards Institute) guidelines as previously described by Draper et al. (2013) with some modifications. Briefly, each well within the microtitre plate was treated with phosphate buffered saline (PBS) containing 1% (w/v) bovine serum albumin (PBS/BSA) (200 μl) and incubated for 30 min at 37°C. Following this, the wells were washed with PBS (200 μl) and allowed to dry. Overnight cultures of *L. monocytogenes* F6854 were then sub-cultured into fresh broth and grown to an OD_600_ of 0.5 [∼10^8^ colony forming units (cfu)/ml] and subsequently diluted to a final concentration of ∼10^5^ cfu/ml. Stock concentrations of nisin A (100 μg/ml), nisin M21A (128 μg/ml), Nisaplin^®^ (25 mg/ml), and citric acid (150 μg/ml) were prepared in TSB, while 99%-cinnamaldehyde (10 mg/ml) was prepared in a 50% ethanol solution. Each antimicrobial was subjected to twofold serial dilutions in TSB in the PBS/BSA treated plate and the appropriately diluted F6854 strain was subsequently added (100 μl). 96 well microtitre plates were incubated overnight at 37°C. Each MIC was performed in triplicate. The MIC was determined as the lowest concentration at which there was no visible growth.

### Growth Curves

Growth curves were performed in a checkerboard assay manner in 96 well microtitre plates. A twofold serial dilution of either nisin A or nisin M21A were performed along the abscissa excluding the final row. In a separate 96 well microtitre plate, serial twofold dilutions of the antimicrobials sodium acetate, sodium benzoate (both food grade, Sigma), citric acid, potassium sorbate, carvacrol, lactoferrin and 99% *trans*-cinnamaldehyde (hereafter referred to as cinnamaldehyde) (all research grade, Sigma) and Nisaplin^®^ (Danisco, DuPont Nutrition and Health) were performed separately along the ordinate excluding column 1. These dilutions were directly transferred into the corresponding wells on the original plate. 100 ml of a 2% inoculum from an overnight strain was added to each well on the plate. Cell growth was measured spectrophotometrically over a 24 h period using MWGt Sirius HT plate reader (BioTek Instruments, USA).

### Biofilm Growth

Biofilm growth was performed as previously described by Field et al. (2015a), but with modifications as follows. TSB supplemented with 1% D-(+)-glucose (Sigma) (TSBg) was used in order to aid biofilm formation (Choi et al., 2013). 200 μl of TSBg containing a final concentration of 2% inoculum from an overnight strain was added to test wells of a sterile PBS/BSA treated 96 well microtitre plate (Starstedt, Germany). Additionally, 200 μl of TSBg was added to control wells as a negative control. Plates were incubated at 37°C for 48 h to allow for biofilm attachment and formation.

### Established Biofilm Treatment

Biofilm inhibition assays were carried out as previously described by Field et al. (2015b). Here, nisin A, nisin variant M21A, Nisaplin^®^, citric acid and 99% cinnamaldehyde were used to treat *L. monocytogenes* F6854 biofilms. By combining a variety of concentrations of the aforementioned antimicrobials, the minimum amounts needed in combination for effective treatment of an F6854 biofilm was established as follows; 100 μl of the appropriate concentration for each peptide and/or antimicrobial in TSB were added to the biofilm, both alone and in combination. TSB alone was added to a set of wells as a negative control. A number of biofilm containing wells (labeled untreated) and wells containing TSB alone (labeled control) were left untreated and the plate was incubated at 37°C for 24 h. In order to determine the metabolic activity of the biofilm after antimicrobial treatment an XTT assay was performed as previously described by Field et al. (2015b). The principle of the XTT assay is that viable cells will reduce the tetrazolium salt XTT [2,3-bis(2-methyloxy-4-nitro-5-sulfophenyl)-2H-tetrazolium-5 carboxanilide] (Sigma) to an orange-colored water-soluble product measurable via a standard microplate absorbance reader at OD_492 nm_ (Tunney et al., 2004). Briefly, all test and control wells were washed with 200 μl sterile PBS. 100 μl of a solution containing 500 mg/l XTT sodium salt and 10 μM menadione (Sigma) was added to each well. The plates were incubated in darkness at 37°C for 2 h. Absorbance was read at 492 nm.

### Statistical Analysis

All aforementioned growth curve experiments were conducted in triplicate. Six replicates of each established biofilm treatment experiment were performed. Statistical analysis was performed using GraphPad Prism 6, GraphPad Software, Inc. Unless otherwise stated a one-way ANOVA was performed followed by Tukey test, where *P*-values are denoted as: ^∗^*P* > 0.05; ^∗∗^*P* ≤ 0.01; ^∗∗∗^*P* ≤ 0.001.

## Results

### Deferred Antagonism Agar Diffusion Assay

**FIGURE 1:**
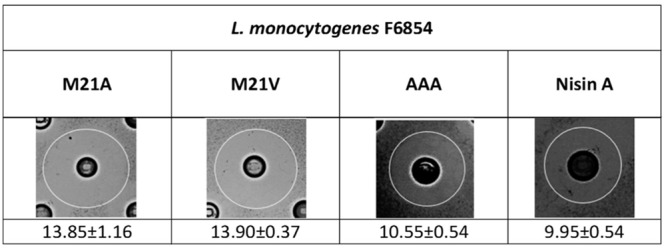
**Agar diffusion assays depicting the antimicrobial effects of *Lactococcus lactis* NZ9800 strains producing the mutants M21V, M21A, AAA and nisin A (wild type) against *Listeria monocytogenes* F6854.** Diameter zone sizes (mm) are the average of triplicate experiments.

In order to evaluate the antimicrobial activity of nisin A and its derivatives against *L. monocytogenes* F6854, a selection of nisin variant producers were assessed via deferred antagonism agar diffusion assay. Of the variant strains tested the observed zone sizes for three derivative producers, AAA (where the three hinge amino acids in positions 20–22 have been replaced with alanines), M21A and M21V were consistently enhanced when compared to the corresponding isogenic nisin A producer (**Figure 1**). To date a number of studies have been conducted that have highlighted the efficacy of M21V against strains of *L. monocytogenes* (Field et al., 2008, 2015a; Campion et al., 2013). Here, we noted that the antimicrobial efficiency of the M21A producer appeared equivalent to that of M21V, while they both had a greater degree of activity against the indicator strain than that of the producer of the AAA derivative. As a result of these observations the purified nisin M21A peptide was chosen for further investigation *via* specific activity studies/growth curve experiments.

### Minimum Inhibitory Concentrations

The activities of purified nisin A, nisin M21V and M21A were assessed using broth based MIC assays (**Table 1**). The MIC recorded for nisin A against *L. monocytogenes* F6854 was 12.5 μg/ml, a result that is consistent with previously published results for *L. monocytogenes* EGDe::pPL2*lux*pHELP (Campion et al., 2013). The MIC for both nisin M21V and M21A was 6.25 μg/ml, i.e., half that observed for nisin A. Indeed, it has previously been established that nisin M21V has twofold greater activity than nisin A against a number of *L. monocytogenes* (Field et al., 2010a; Molloy et al., 2012). In addition to nisin and its variants, the antimicrobial activity of citric acid, sodium acetate, sodium benzoate, potassium sorbate, carvacrol, lactoferrin, cinnamaldehyde and Nisaplin^®^ against F6854 were also assessed (**Table 1**).

**Table 1:** Minimum inhibitory concentrations of nisin peptides and antimicrobials required to treat *L. monocytogenes* F6854.

Antimicrobial	MIC (μg/ml)
Nisin A	12.5
Nisin V	6.25
M21A	6.25
Citric Acid	2340
99% cinnamaldehyde	625
Lactoferrin	>2500
Sodium acetate	250,000
Sodium benzoate	25,000
Carvacrol	310
Potassium sorbate	37,500
Nisaplin^®^	393

### Dual Antimicrobial Inhibition of *L. monocytogenes* F6854

**FIGURE 2:**
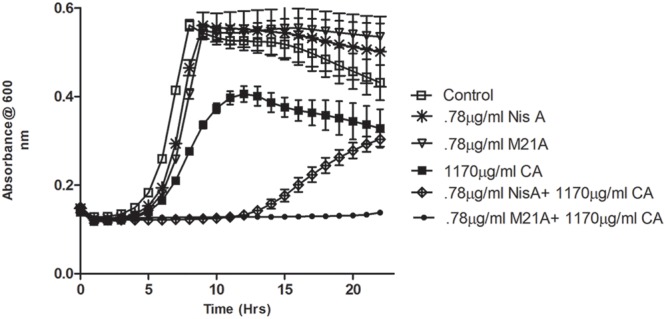
**Growth curve depicting the effect of both M21A and Nisin A (Nis A) alone and in combination with citric acid (CA) against *L. monocytogenes* F6854.** Growth was measured via triplicate readings at OD_600 nm_ over a period of 22 h.

**FIGURE 3:**
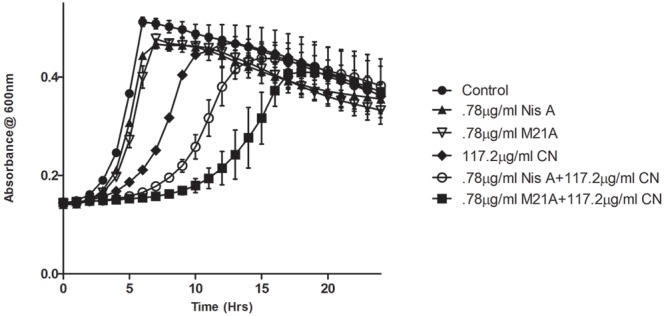
**Growth curve depicting the effect of both M21A and Nisin A (Nis A) alone and in combination with cinnamaldehyde (CN) against *L. monocytogenes* F6854.** Growth was measured at OD_600 nm_ over a period of 24 h. Results were recorded in triplicate.

Preliminary checkerboard assays combining nisin A and M21A with each of the other, aforementioned, antimicrobials were performed in order to determine if any of the combinations revealed enhanced antimicrobial activity. While many combinations showed marginally enhanced effects (data not shown), the combination of nisin A, M21A and either citric acid or cinnamaldehyde showed the most dramatic results. Therefore, further triplicate growth curve experiments were conducted using both nisin A and M21A in combination with either citric acid or cinnamaldehyde against *L. monocytogenes* F6854 over a 24 h period (**Figures 2** and **3**). At 0.78 μg/ml, neither nisin peptide inhibited the pathogen. Citric acid, at 1170 μg/ml, extended the lag phase by approximately 1 h and reduced the final OD. Combining these concentrations of nisin A and citric acid extended the lag phase by 14 h and an OD_600 nm_ of 0.3 was only reached after 22 h. When citric acid (1170 μg/ml) was combined with M21A (0.78 μg/ml), complete inhibition of growth was observed (**Figure 2**). An enhanced specific activity of M21A in comparison to nisin A was also noted during combinatorial studies with cinnamaldehyde against *L. monocytogenes* F6854 (**Figure 3**). 117.2 μg/ml of cinnamaldehyde alone delayed the lag phase by approximately 2 h but, when combined with M21A (0.78 μg/ml) the lag phase was extended to 11 h, approximately 4 h longer than that observed for nisin A and cinnamaldehyde.

### Biofilm Treatment

**FIGURE 4:**
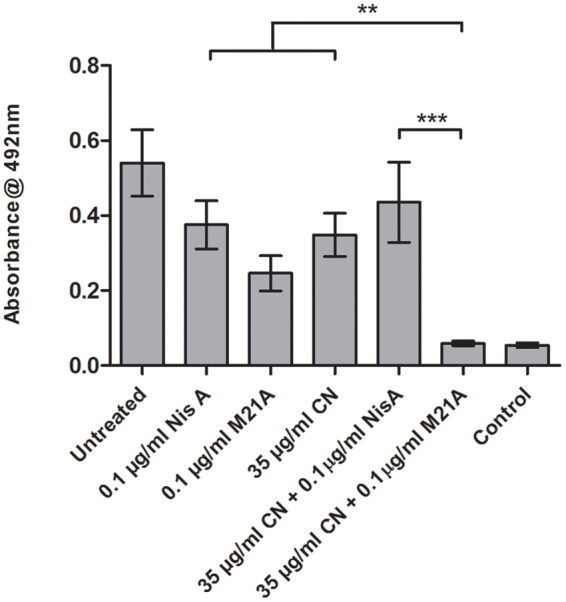
**Treatment of an established F6854 biofilm – NisA and M21A in combination with cinnamaldehyde.** Treatment of established (48 h) biofilms of *L. monocytogenes* F6854 with M21A, nisin A (Nis A) and cinnamaldehyde (CIN) alone and in combination. Treatment was performed in triplicate over 24 h. Graph indicates the level of metabolically viable cells remaining following treatment as determined through XTT assay. ^∗∗^*P*-value ≤ 0.01; ^∗∗∗^*P*-value ≤ 0.001.

**FIGURE 5:**
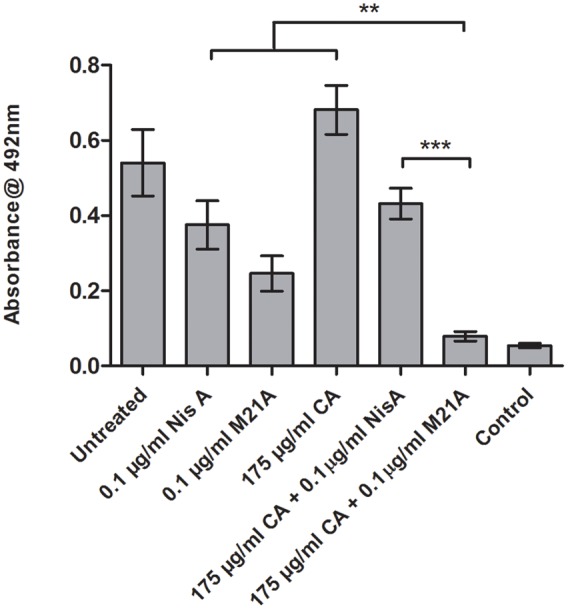
**Treatment of an established F6854 biofilm – NisA and M21A in combination with citric acid.** Treatment of established (48 h) biofilms of *L. monocytogenes* F6854 with M21A, nisin A (Nis A) and citric acid (CA) alone and in combination. Treatment was performed in triplicate over 24 h. Graph indicates the level of metabolically viable cells remaining following treatment as determined via XTT assay. ^∗∗^*P*-value ≤ 0.01; ^∗∗∗^*P*-value ≤ 0.001.

These combinations were examined to determine if they also demonstrated enhanced efficacy against established F6854 biofilms (**Figures 4** and **5**). It was observed that for biofilm treatment the ratio (μg/ml: μg/ml) of cinnamaldehyde or citric acid to nisin A or M21A was in some case 150 times the ratio used for planktonic growth inhibition (**Table 2**). Cinnamaldehyde (35 μg/ml) and M21A (0.1 μg/ml) showed significantly enhanced antimicrobial effects against the established biofilm compared to the corresponding combinations of cinnamaldehyde and nisin A. Biofilm treatment with citric acid (175 μg/ml) and M21A (0.1 μg/ml) in combination reduced the viable cell numbers significantly compared to the antimicrobial/peptide alone (**Figure 5**). Most striking was the significant fivefold difference observed between the nisin A-citric acid combination and the dual effect of M21A and citric acid. Notably, when used at these concentrations the impact of the nisin derivative M21A in combination with either citric acid or cinnamaldehyde was statistically indistinguishable from the non-biofilm control, indicating complete eradication of the biofilm.

**Table 2:** Ratio of antimicrobials used in combination to treat *L. monocytogenes* F6854 in both planktonic culture and as established biofilms.

Antimicrobial combinations	Planktonic culture		Established biofilm	
	(μg/ml: μg/ml)	Ratio	(μg/ml: μg/ml)	Ratio
Nisin A: citric acid	0.78:1170	1:150	0.1:175	1:1750
Nisin A: cinnamaldehyde	0.78:117.2	1:150^∗^	0.1:35	1:350
M21A: citric acid	0.78:1170	1:150	0.1:175	1:1750
M21A: cinnamaldehyde	0.78:117.2	1:150^∗^	0.1:35	1:350

*These ratios are based on the minimum concentration (μg/ml) of each antimicrobial required in combination to inhibit F6854.*

*^∗^Complete inhibition of *L. monocytogenes* was not achieved in this case.*

### Inhibitory Effect of Nisaplin^®^ in Combination with Antimicrobials on F6854 Biofilm

**FIGURE 6:**
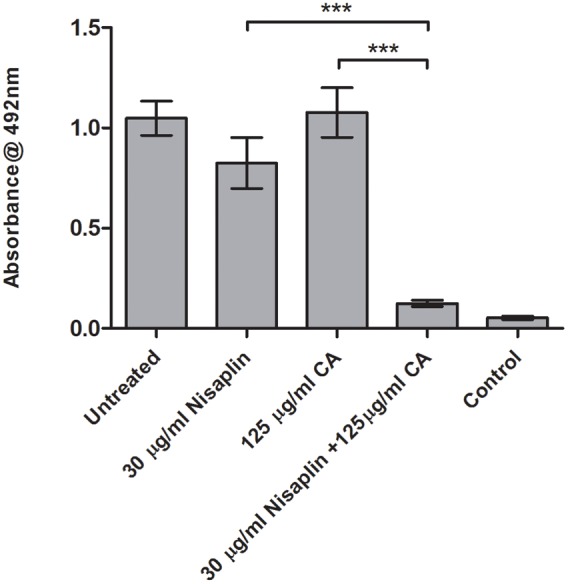
**Treatment of an established F6854 biofilm – Nisaplin^®^ and cinnamaldehyde.** Treatment of established (48 h) biofilms of *L. monocytogenes* F6854 with Nisaplin^®^ and cinnamaldehyde (CN) alone and in combination. Treatment was over 24 h and performed in triplicate. Graph indicates the level of metabolically viable cells remaining following treatment as determined through XTT assay. ^∗∗∗^*P*-value ≤ 0.001.

**FIGURE 7:**
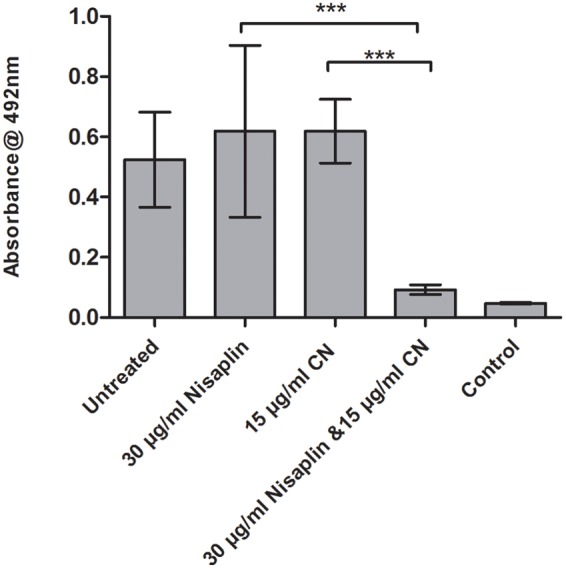
**Treatment of an established F6854 biofilm – Nisaplin^®^ and citric acid.** Treatment of established (48 h) biofilms of *L. monocytogenes* F6854 with Nisaplin^®^ and citric acid alone and in combination. Treatment was over 24 h and performed in triplicate. Graph indicates the level of metabolically viable cells remaining following treatment as determined via XTT assay. ^∗∗∗^*P*-value ≤ 0.001.

It was decided to investigate if inhibitory effects were achievable using citric acid and cinnamaldehyde in combination with a readily available nisin A containing food additive Nisaplin^®^. With the enhanced inhibitory effects of M21A over nisin A evident in the previous combination studies, a greater amount of nisin A and hence Nisaplin in combination would be required to cause eradication of an established biofilm. This would suggest that production of a derivative of Nisaplin^®^-equivalent containing M21A would enhance the antimicrobial potency of these additives. Since Nisaplin^®^ contains only 2.5% nisin it was of interest to confirm that the remainder of the ingredients within this additive (or a potential M21A containing product) would not inhibit the observed synergy with citric acid and cinnamaldehyde. As seen in **Figure 6** a presumptive synergistic effect was observed between Nisaplin^®^ and cinnamaldehyde against an established F6854 biofilm (**Figure 6**). Neither Nisaplin^®^ [30 μg/ml (nisin A content: 0.75 μg/ml)] nor cinnamaldehyde (15 μg/ml) alone had a statistically significant effect on the biofilm in relation to the untreated control. However, the dual effect of both antimicrobials at the same concentrations showed a statistically significant decrease in the biofilm. A similar result was observed when Nisaplin^®^ (30 μg/ml (nisin A content: 0.75 μg/ml)) and citric acid (125 μg/ml) were combined (**Figure 7**). Neither Nisaplin^®^ nor citric acid alone had a statistically significant effect on the biofilm. However, the combination of both antimicrobials at the same concentrations showed a statistically significant (*P* ≤ 0.001) decrease in biofilm viability. When we consider the nisin A content of the Nisaplin^®^ used (30 μg/ml) in these combinations it corresponds to 0.75 μg/ml (2.5%). This is >7 times the amount of purified M21A peptide utilized in the corresponding combinations (**Figures 4** and **5**) to achieve biofilm eradication. It must be noted however that the levels of citric acid and cinnamaldehyde utilized were slightly decreased (1.4 and 2.3 times respectively) in comparison to the aforementioned M21A containing biofilm treatments.

## Discussion

Listeriosis, the disease associated with the ubiquitous food borne pathogen *L. monocytogenes*, has a low incidence rate (Maertens de Noordhout et al., 2014), but an extremely high mortality rate (20–30%) (Laksanalamai et al., 2012) and is rigorously monitored within the food and beverage industry. Microbial standards within the EU state that <100 cfu/g *L. monocytogenes* is permitted in Ready-To-Eat (RTE) foods that do not have the potential to support listerial growth throughout their shelf life. However, in infant foods, foods for medical purposes and RTE foods that do support listerial growth, the micro-organism must be absent in any 25 g sample at the time of production (The Commission of the European Communities, 2005). The US maintain a zero tolerance policy on *L. monocytogenes* in any RTE product (And and Hoover, 2003). Due to these highly strict regulations, discovering novel and safe means to control of *L. monocytogenes* is of significant importance and benefit to food manufacturers.

The main focus of this study was to determine if an enhanced effect could be obtained by combining a derivative of nisin with other food-grade antimicrobials to inhibit the growth of *L. monocytogenes* F6854 in liquid culture and, in addition, to eradicate established biofilms of this same strain. By including bioengineered versions of nisin in this study we sought to and achieved increased antimicrobial potency. Due to the documented sensitivity of *Listeria* to nisin and the relevance of both to the food industry (Thomas and Wimpenny, 1996; Arqués et al., 2008; Pinto et al., 2010; Field et al., 2015a), strain F6854, which has been linked to contaminated turkey frankfurters, represented an ideal target for nisin and food-grade antimicrobial combinations. The food additives sodium benzoate, sodium acetate, potassium sorbate, lactoferrin, carvacrol, citric acid and cinnamaldehyde were all tested initially. We noted that the amount of sodium acetate required for inhibition was extremely high as compared to the other antimicrobials in this study. The MIC obtained for both sodium benzoate and potassium sorbate are higher than that previously reported for other *L. monocytogenes* strains (Neetoo et al., 2008). In addition, the concentration of cinnamaldehyde required to inhibit the indicator strain was found to be 625 μg/ml. This is approximately 50% greater than the MIC previously published for cinnamaldehyde against a number of different *L. monocytogenes* strains. The MIC for carvacrol was, however, in keeping with results targeting two strains from this same study (Field et al., 2015a). Following initial broth-based growth curves the most notable effects were observed however when citric acid or cinnamaldehyde were used in combination with nisin.

Citric acid has a long history of use in food and has GRAS status (Lemmerer and Bernstein, 2010). It is used for its antimicrobial properties, which includes its ability to function as a chelator (Brul and Coote, 1999) and to pass freely through the cell membrane altering the cytoplasmic pH (Cotter and Hill, 2003). It is often used in combination with other preservatives and/or antimicrobials to inhibit pathogens in food. Indeed, the use of citric acid has proven effective in combination with nisin against Gram-negative bacteria (Phillips, 1999) and in combination with nisin against a strain of *Listeria innocua* (Al-Holy et al., 2006). Less is known about the effect of citric acid on *L. monocytogenes*. Cinnamaldehyde has previously been shown to have an antimicrobial effect on strains of *L. monocytogenes* (Gill and Holley, 2004). Although not fully elucidated, cinnamaldehyde is believed to function through inhibition of glucose uptake, inhibition of enzymes involved in cell function and disruption of the cell membrane (Gill and Holley, 2004; Hyldgaard et al., 2012). Furthermore, a recent study by Field et al. (2015a) also demonstrated that cinnamaldehyde in combination with a nisin variant was more effective than the essential oil or variant alone against a strain of *L. monocytogenes* in planktonic form.

Following the selection of two antimicrobial compounds, cinnamaldehyde and citric acid, we investigated the consequences of their use in combination with nisin A or a bioengineered derivative thereof. From a number of nisin A variants tested, both M21A and M21V demonstrated equally enhanced activity compared to the wild type in both MIC and agar diffusion assays. Nisin A derivatives M21A and M21V both contain a single amino acid changes at position 21. In the case of M21A, a methionine is replaced by an alanine and in M21V, the same methionine is replaced by a valine. M21V has previously demonstrated enhanced activity against strains of *L. monocytogenes* and has been documented in numerous studies (Field et al., 2010b, 2015a; Campion et al., 2013; Healy et al., 2013) and so it was included in MIC studies in order to compare its activity with that of M21A, which until now remained relatively undocumented. This is the first study in which the MIC of the bioengineered derivative M21A has been tested against a strain of *L. monocytogenes*. Having established that the antimicrobial efficacy of M21A against *L. monocytogenes* F6854 was twofold greater than that of nisin A, we went further to demonstrate that M21A possesses enhanced ability when compared to the wild type with respect to eradicating formed *L. monocytogenes* biofilms, a characteristic not attributed to any other bioengineered nisin derivative to date.

Initially inhibition of bacterial growth utilizing the chosen food-grade additives (citric acid and cinnamaldehyde) and lantibiotic peptides nisin A and M21A were investigated using broth based growth curve assays. These studies demonstrated that complete inhibition of this strain of F6854 could be obtained using M21A in combination with citric acid, while nisin A at the same concentrations was unable to achieve such potency. Although a less dramatic result was obtained from the combination of M21A and cinnamaldehyde, M21A exhibited a greater inhibitory effect against *L. monocytogenes* F6854 both alone and in combination with cinnamaldehyde. Hence, the anti-listerial properties of M21A can be further enhanced with the addition of citric acid or cinnamaldehyde. This led to investigation to determine if these enhanced effects could also inhibit established biofilms of the same strain.

Nisin has been used in previous studies with a view to inhibiting bacterial biofilms (Mahdavi et al., 2007; Gou et al., 2011). In addition, nisin in combination with antimicrobials has also been investigated in relation to the inhibition of bacterial biofilms and more recently the use of a nisin derivative has been investigated in relation to the inhibition of a *Staphylococcus* biofilm (Field et al., 2015b). However, the current study is the first to our knowledge to show the antimicrobial effects of a bioengineered version of nisin (both alone and in combination) on *L. monocytogenes* biofilms. The demonstrated enhanced antimicrobial effect of M21A in combination with food grade preservatives, citric acid or cinnamaldehyde, resulted in a reduction in viable cells that could not be statistically distinguished from a non-biofilm control.

When we examined the concentrations of antimicrobials required to obtain inhibition we observed the same trend for planktonic and biofilm experiments, with a low level of nisin and a greater amount of citric acid/cinnamaldehyde required. When we examine the ratio of antimicrobial concentrations used for biofilm inhibition the level of cinnamaldehyde and citric acid required to treat the biofilms is up to 150 times that required for planktonic cells. This confirms that determining the ideal proportion of each antimicrobial is vital for optimal synergistic effect and is dependent on the physiological state of bacteria whether it be planktonic or existing as a biofilm. It is also possible that the structural properties of the extracellular polymeric matrix or subtle differences in the behavior of cells within the biofilm occur in comparison to their planktonic counterparts. In fact, it has been shown that lower levels of Nisaplin^®^ are required to inhibit *L. monocytogenes* within a food matrix when compared to broth based assays (Hara et al., 2009).

Currently, nisin A-containing products such as Nisaplin^®^ are commercially available to be used as food preservatives. Here, we show that Nisaplin^®^-citric acid/cinnamaldehyde combinations could significantly reduce and in fact eradicate *Listeria* biofilms. However, >7 times more of the antimicrobial component (i.e., nisin A) was required in comparison to the M21A purified peptide counterpart. Nonetheless due to market availability of Nisaplin^®^ and these food additives, such combinations are readily applicable and may even present a lower cost alternative to the control of *L. monocytogenes* within the food industry. Although, nisin sensitivity to *L. monocytogenes* has been documented, the development of nisin resistant mutants has been reported (Gandhi and Chikindas, 2006). Therefore, the combinations within this study represent an example of hurdle technology whereby bacterial growth struggles to overcome the potency of dual acting antimicrobials. In addition, by combining these antimicrobials it allows for the use of lower concentrations of the individual additives in food items. This may prove beneficial in relation to the use of the essential oil cinnamaldehyde – the use of which has been limited due to the amount needed for an antimicrobial effect affecting the overall organoleptic properties of the food itself. However, sensory evaluation of a chosen food containing this level of the essential oil or citric acid should be conducted in order to distinguish if there are any negative effects to the organoleptic properties of the food.

Ultimately, this study has identified a clear application for M21A in combination with either citric acid or cinnamaldehyde to inhibit listerial growth, eradicate formed biofilms and hence potentially inhibit biofilm formation. This effect that significantly exceeded the effect of nisin A in the corresponding combination. This study documents the first report of a bioengineered derivative of nisin used with other food additives to control *Listeria* biofilms. We propose that substituting M21A into nisin A-containing products, such as Nisaplin^®^, would further enhance the antimicrobial potency of such products. Ultimately, such combinations should enhance the microbial safety of foods as well as provide consumers with an effective natural alternative to artificial preservatives.

## Author Contributions

Conceived and supervised the study: LD, PC, CH, and RR. Designed the experiments: MS, LD, and P-JH. Performed the experiments: MS and P-JH. Analyzed the data: MS, LD, P-JH, PC, RR, and CH. Wrote the paper: MS, LD, P-JH, PC, RR, and CH.

## Conflict of Interest Statement

The authors declare that the research was conducted in the absence of any commercial or financial relationships that could be construed as a potential conflict of interest.
